# Chicken Egg Yolk Antibody (IgY) Protects Mice Against Enterotoxigenic *Escherichia coli* Infection Through Improving Intestinal Health and Immune Response

**DOI:** 10.3389/fcimb.2021.662710

**Published:** 2021-04-13

**Authors:** Shuaijuan Han, Yang Wen, Fengfan Yang, Pingli He

**Affiliations:** ^1^ State Key Laboratory of Animal Nutrition, College of Animal Science and Technology, China Agricultural University, Beijing, China; ^2^ College of Animal Science and Technology, Hebei Agricultural University, Baoding, China; ^3^ Hubei Shendi Biological Technology Co., LTD, Jingmen, China

**Keywords:** egg yolk antibody, IgY, enterotoxigenic *Escherichia coli*, intestinal health, immune response

## Abstract

Chicken egg yolk antibody (IgY), considered as a potential substitute for antibiotics, has been used for preventing pathogens infection in food, human and animals. This study investigated effects of IgY on growth, adhesion inhibitory and morphology of enterotoxigenic *Escherichia coli* (ETEC) K88 *in vitro*, and evaluated the protective effects of IgY on intestinal health and immune response of mice infected with ETEC *in vivo*. Sixty pathogen-free C57BL/6J (4-6 weeks of age) mice were divided into six treatments: control (neither IgY nor ETEC infection), ETEC infection, ETEC-infected mice treated with 250 μL of high-dose (32 mg/mL), medium-dose (16 mg/mL) or low-dose (8 mg/mL) anti-ETEC IgY, or ETEC-infected mice treated with 250 μL of non-specific IgY (16 mg/mL). Anti-ETEC IgY inhibited ETEC growth, reduced adherence of ETEC to intestinal epithelial cells J2 and damaged the morphology and integrity of ETEC cell. Oral administration of anti-ETEC IgY effectively ameliorated ETEC-induced clinical signs, reduced ETEC colonization and intestinal permeability, alleviated inflammatory response through reducing the production and expression of proinflammatory cytokines, improved intestinal morphology, and inhibited excessive activation of the mucosal immune response of challenged mice. The overall protective effects of high-dose and medium-dose anti-ETEC IgY against ETEC infection were more effective. These results suggest that anti-ETEC IgY may function as a promising novel prophylactic agent against enteric pathogens infection.

## Introduction

Enterotoxigenic *Escherichia coli* (ETEC) cause serious health problems for young animals and human infants due to contaminated food or water. ETEC cause diarrhea, intestinal inflammatory diseases and alter intestinal microbial diversity by adhering to intestinal epithelial cells, secreting enterotoxin and releasing lipopolysaccharide (Fleckenstein et al., 2010; Smith et al., 2010). In practice, antibiotics are currently used to prevent and treat pathogens, but long-term application will cause negative effects such as bacterial resistance, imbalance of intestinal flora in animals and drug residues, which seriously threaten animal and human health (Thacker, 2013). Hence, finding safe and effective antibiotic alternatives to prevent ETEC infection is an urgent and significant task in animal husbandry.

Chicken IgY, known as the immunoglobulin Y, is an antigen-specific antibody produced by B lymphocytes and accumulated in the yolk of chicken eggs. IgY as a bioactive constituent has been used for preventing pathogens infection in food, human and animals. Compared with other immunoglobulins obtained from mammalian animals, chicken IgY has several advantages, including: (a) IgY production process is hygienic, non-invasive, cost-efficient and convenient; (b) IgY does not cause an adaptive immune response of animals (Nilsson et al., 2007); (c) IgY neither activate complement nor recognized by intestinal epithelial Fc-receptors in mammals (Silva and Tambourgi, 2010; Kovacs-Nolan and Mine, 2012); (d) IgY shows high avidity and antigen-specificity and is extraordinarily stable (Kovacs-Nolan and Mine, 2012). Thus, IgY has attracted much attention for passive immunization (Baloch et al., 2015; Li et al., 2015; Abbas et al., 2019). Numerous studies have shown that specific IgY has positive effects in the prevention and treatment of pathogens including rotaviruses, coronavirus, epidemic diarrhea virus and transmissible gastroenteritis virus (Diraviyam et al., 2014; Lee et al., 2015). Another study reported that anti-*Salmonella* IgY significantly reduced the number of *Salmonella* in feces and cecal contents, and reduce morbidity and mortality of infected chickens (Rahimi et al., 2007). Several possible mechanisms of action have been proposed, including: (i) IgY causes pathogen agglutination; (ii) inhibition of pathogen adhesion to cells; (iii) neutralization of toxins; (iv) regulation of phagocytosis by phagocytes and modulation of body immunity to provide passive immune protection for young animals (Zhou and Ma, 2018; Xu et al., 2019). In our previous study, we found that hyperimmunized egg yolk powder could reduce diarrhea incidence and *E. coli* numbers in feces, ameliorate negative effects of *E. coli* on intestinal health indices, and enhance immunity of weaned pigs (Han et al., 2019). However, the exact mechanisms of biological function to affect the health status of humans and animals are still unclear. Furthermore, few studies have determined the intestinal health and immune-modulatory effects of specific IgY on the host.

Given that ETEC cause serious health problems for young animals and human infants and the current use of antibiotics is tightly restricted, new antibacterial strategies are urgently needed. Therefore, the objective was to evaluate whether IgY exerts obvious anti-bacterial activity *in vitro*. A second objective was to explore potential uses and possible mechanisms of IgY in preventing ETEC infection in a mouse model of intestinal inflammation. This study provides a theoretical reference for IgY in protecting animals from bacterial diseases such as ETEC infection.

## Materials and Methods

### Bacteria and Preparation of IgY

ETEC K88 (BNCC 125988) were purchased from Beijing Beina Chuanglian Biotechnology Institute (Beijing, China). Bacterial culture conditions were performed according to our previous study (Han et al., 2019). The cell suspension (2.5 × 10^9^, 5 × 10^10^ colony forming units (CFU)/mL) was used for the immunization of laying hens and mice infection, respectively. Anti-ETEC K88 IgY was obtained according to the method in our previous study (Han et al., 2019).

### Growth Inhibition Test

The ETEC K88 (1.0 × 10^7^ CFU/mL) was grown in Luria-Bertani medium including a range of density (0, 1.25, 2.5 and 5 mg/mL) of sterilized anti-ETEC K88 IgY, which was filtered using a 0.22 μm filter. Non-specific IgY (5 mg/mL) was performed as the negative control. These mixtures were incubated at 37°C with shaking at 200 rpm. Suspensions were collected and measured at OD_600_ nm at 2-h intervals with three biological replicates. Then, growth inhibition curves of IgY were measured by plotting the value of OD_600_ nm against time.

### Cell Culture and Adherence Assay

Intestinal epithelial cells J2 (IPEC-J2) were kindly donated by Dr. Shiyan Qiao (China Agricultural University, China). Cells were grown in Dulbecco’s Modified Eagle’s medium/Ham’s nutrient mixture F-12 (DEME/F12) containing 1% streptomycin (10,000 g/mL)/penicillin (10,000 U/mL), 10% fetal bovine serum, 1% insulin/transferrin/selenium (ITS) and 5 µg/L epidermal growth factor. Cells were cultured in a 95% humidified atmosphere with 5% CO_2_ at 37°C.

IPEC-J2 cells (1 × 10^6^ cells per well) were seeded onto 6-well plates and incubated in medium without antibiotics to about 80% confluence. Before infection, ETEC K88 (2 × 10^8^ CFU/mL) was pre-incubated with 5 mg/mL anti-ETEC K88 IgY, 5 mg/mL non-specific IgY and PBS at 37°C for 1 h, respectively. Then, pre-incubated ETEC K88 cells were used for infection in IPEC-J2 cells (n = 3) for 2 h in 5% CO_2_ atmosphere at 37°C. Unbound bacteria were removed by three washings with sterile PBS. IPEC-J2 cells were lysed with 1 mL of 0.1% Triton X-100 for 5-10 min, serially diluted, plated onto MacConkey-agar plates and incubated at 37°C for 16 h. Initial and final bacterial colonies were counted.

### Transmission Electron Microscopy

To evaluate the alteration of ETEC K88 cell ultrastructure and membrane morphology, transmission electron microscopy (TEM) was performed as described previously (Wang et al., 2017), with minor modifications. Briefly, ETEC K88 cells (10^7^ CFU/mL) were incubated with 5 mg/mL of sterilized IgY. The negative and blank controls included 5 mg/mL of non-specific IgY and sterile PBS, respectively. After centrifugation, bacterial cells were fixed with 2.5% (v/v) glutaraldehyde in PBS (0.1 mol/L, pH 7.4) at 4°C for 2-4 h, covered with 1% agarose, and washed with PBS three times. Cells were then fixed with 1% osmium tetroxide at room temperature for 2 h, dehydrated with acetone, and embedded in Epon. Thin sections were stained with uranium acetic acid and lead citrate for 15 min and observed by using TEM (Model JEM-1230, JEOL, Japan).

### Mice

All mouse experiments were approved and performed according to the guidelines of the Institutional Animal Care and Use Committee at China Agricultural University (Beijing, China). Pathogen-free, female C57BL/6J mice (4-6 weeks of age, 17.12 ± 0.39 g initial body weight) were obtained from Beijing HFK Bioscience Co., Ltd. (Beijing, China). All mice were housed in the same humidity- and temperature-controlled room with 12 h light/dark cycle. All mice were kept in a laminar flow cabinet individually and feed and water were provided ad libitum.

### Mice Infection Model

Mice (n=60) were divided randomly into 6 treatments (10 mice per treatment) according to their body weight after a 3-day adaptation period. Treatments were as follows: uninfected control (neither IgY nor ETEC K88 infection), ETEC K88 infected control, mice challenged with ETEC K88 and treated with 250 μL of high-dose (32 mg/mL), medium-dose (16 mg/mL) or low-dose (8 mg/mL) anti-ETEC IgY or 250 μL of non-specific IgY (16 mg/mL). Prior to experimental infection, mice in the control and ETEC group were pre-treated with 250 μL of sterile PBS by gavage, whereas mice in test groups were pre-treated with 250 μL of a solution containing 32, 16 or 8 mg/mL of anti-ETEC IgY, or 16 mg/mL of non-specific IgY once daily by oral gavage for 5 consecutive days. The following day, except for the control group, all mice were inoculated orally with 300 μL of ETEC K88 (5×10^10^ CFU/mL). Mice were fasted (feed and water) for 4 h before challenge, and deprived of feed and water for 3 h after challenge. After challenge, mice in the control and ETEC group continued to be treated with sterile PBS by oral gavage, whereas mice in test groups continued to receive their assigned doses of IgY for 3 d.

After challenge, clinical signs of mice were observed daily using a scoring system that was reported previously (Warren et al., 2013; Pizarro-Guajardo et al., 2017). Stool scores were as follows: 0, normal stool (dry stool, intact granules); 1, mild diarrhea stool (slight sticky stool, adhesion to filter paper); 2, moderate diarrhea stool (soft stool, no formation of feces, no separation of fecal water); 3, severe diarrhea stool (liquid stool). Mice with a fecal score greater than 1 were considered to have diarrhea. Body weight and survival rate of mice were recorded daily for 4 days after ETEC inoculation.

### Sample Collection and Processing

Fresh fecal samples were taken from six same mice (selected randomly from each group) for each of the 4 days after ETEC K88 challenge. The bacterial load of *E. coli* was measured on MacConkey agar plates (OXOID, Hampshire, UK). A second fecal sample was kept in a freezer at -80°C for analysis of the concentration of short chain fatty acid (SCFA). At day 4 post-infection, blood samples of mice were collected. Serum was obtained by centrifugation (3000×g, 15 min) and stored at -80°C for further analysis.

Intestinal tissues collected from the middle of duodenum, jejunum and ileum were flushed gently with normal saline (0.9% sodium chloride), fixed in 4% paraformaldehyde, and embedded in paraffin for morphological and pathological analysis. Another section of jejunal tissue was collected, snap-frozen in liquid nitrogen, and stored at -80°C for secretory immunoglobulin A (sIgA) analysis. Cecal contents were collected and stored at -80°C for analysis of microbial composition and SCFA content.

### Fecal Bacterial Load and Cecal Microbial Composition Analysis

Fresh fecal samples were collected each day from mice after challenge, resuspended in sterile physiological saline solution, and homogenized. The bacterial load of *E. coli* strain was counted by plating 10-fold serial dilutions of homogenates on MacConkey plates, and incubated at 37°C for 24 h. Results were expressed as log_10_ CFU/g of feces. Total bacteria and *E. coli* numbers in cecal contents were determined by quantitative reverse-transcription PCR (RT-qPCR) method as described in our previous study (Han et al., 2019).

### Short-Chain Fatty Acid Analysis

Concentrations of SCFA in feces and cecal contents were determined by ion chromatography (ICS 3000, Thermo, USA). Samples were thawed and thoroughly mixed before analysis. Thawed sample was weighed (about 80 mg) into 8 mL of double-distilled water. The mixture was incubated in an ultrasonic bath for 20 min, and then centrifuged at 10,000 × g for 15 min. The resulting suspension was diluted (1:8) in distilled water. The extracted sample that was filtered through a 0.22-μm filter (100 μL) was injected into ion chromatography and analyzed using AS11 analytical column (250 mm× 4 mm).

### Serum Biochemical Indices Measurement

An automatic biochemistry analyzer (Hitachi, Ltd., Japan) was used to measure concentrations of serum albumin (ALB), globulin (GLB), albumin to globulin ratio (A/G), total protein (TP), urea nitrogen (UN), alanine aminotransferase (ALT) and aspartate aminotransferase (AST) according to commercial kits and manufacturer’s instructions. All the kits were obtained from Biosino Biotechnolgy and Science Inc. (Beijing, China).

### Cytokine, Intestinal Permeability and sIgA Measurement

Serum cytokines, including IL-10, IFN-γ, IL-1β, IL-12p70, IL-2, IL-4, IL-6, IL-5, growth regulates oncogenes (KC/GRO) and TNF-α, were measured using V-PLEX proinflammatory Panel 1 (mouse) kit (Meso Scale Discovery) by a Meso QuickPlex SQ 120 instrument (Meso Scale Discovery) follow the instruction. The mRNA relative expression of *TNF-α*, *IL-10* and *IL-1β* in jejunum of mice were determined using RT-qPCR. The gene for *β-actin* was used as an endogenous reference. The relative expression of genes was expressed using the 2^−ΔΔCt^ (Yu et al., 2020). The primers are listed in **Table S1**.

Concentrations of D-lactate, diamine oxidase (DAO) and immunoglobulins in serum and sIgA in jejunal tissues were determined using corresponding commercially available mouse ELISA kits (Beijing Yonghui Biotechnology Co., Ltd., China) according to procedures described by manufacturers.

### Intestinal Morphology and Histopathology

Random segments of duodenum, jejunum and ileum were excised and fixed with 4% paraformaldehyde, embedded in paraffin, sectioned into 5-μm slices and stained with hematoxylin and eosin (HE) according to standard procedures. Villus height and crypt depth were measured as described previously (Tsirtsikos et al., 2012). The ratio of villus height to crypt depth (V/C ratio) was calculated. Stained sections were observed microscopically by a single scorer blinded to treatments using a scoring system (Barthel et al., 2003; Li et al., 2016). The severity of epithelial cell necrosis, inflammatory cell infiltration, central chylous tube dilatation, and submucosal edema in each intestinal tissue section was assessed according to the following degrees: 0, none;1, mild; 2, gentle; 3, severe.

### Immunohistochemistry of Lymphocyte Populations

Proliferation of intestinal T lymphocytes and subtypes was analyzed according to the conventional method for immunohistochemical analysis (Tu et al., 2016). Briefly, jejunal tissues were fixed in 4% paraformaldehyde, serial dehydrated by a gradient of ethanol, and embedded in paraffin. Sections were dewaxed, rehydrated and washed with PBS, and underwent heat-induced epitope retrieval with preheated epitope retrieval solutions (10 mM citrate buffer, pH 6.0 for CD3 and CD8; EDTA buffer, pH 9.0 for CD4). Endogenous peroxidase activity was quenched and blocked. Sections were incubated at 4°C overnight with corresponding primary mouse monoclonal antibody: mouse anti-CD3 (1:100, Abcam, Cambridge, UK), mouse anti-CD4 (1:200, Santa Cruz Biotechnology, Canada), mouse anti-CD8 (1:200 Abcam, Cambridge, UK). After incubation, sections were washed with PBS and incubated with horseradish peroxidase conjugated goat anti-mouse/rabbit IgG antibody for 50 min at 37°C, developed with diaminobenzidine, and counterstained with hematoxylin. After sealing with neutral gum, sections were observed with a microscope. Negative control was performed without primary antibodies.

### Statistics

Differences in survival rates were analyzed by the chi-square test using SAS (SAS 9.4 software; SAS Inst. Inc., Cary, NC). Other data were analyzed using ANOVA and GLM procedure, and statistical differences among treatments were compared using Student–Neuman–Keuls multiple-range test. Data are expressed as least square means with standard error of the mean (SEM). Statistical significance and tendency were considered as *P* < 0.05 and 0.05 ≤ *P* < 0.10, respectively. *P < 0.05, **P<0.01 and ***P < 0.001.

## Results

### IgY Inhibited the Growth of ETEC

**Figure 1:**
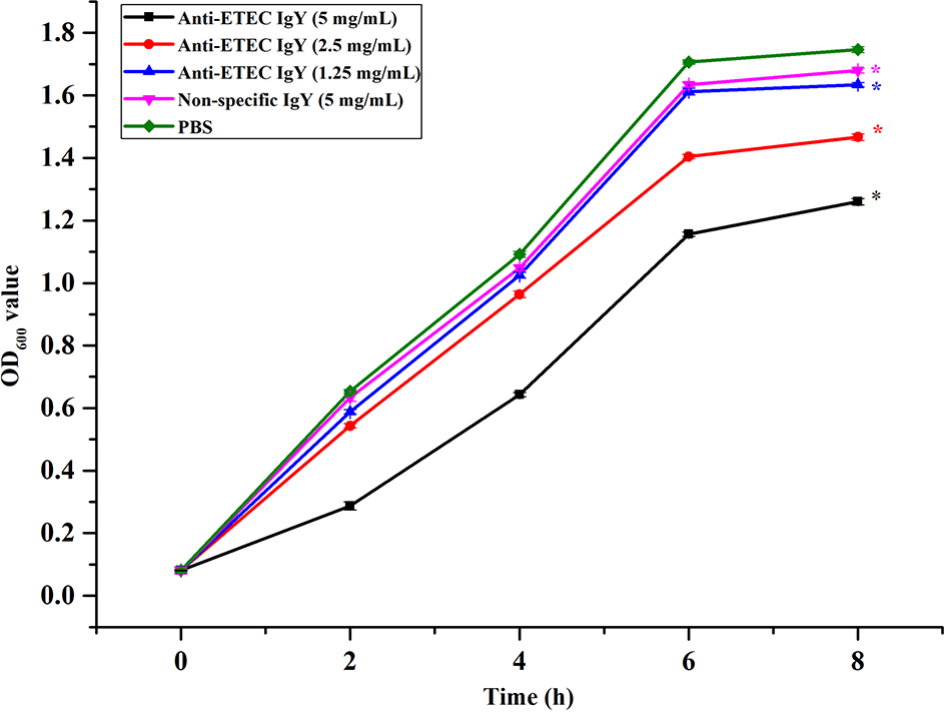
Growth inhibition curves of ETEC K88 treated with different doses of anti-ETEC IgY. Values are expressed as the mean ± SEM (n = 3). *P < 0.05 compared to PBS treatment.

To evaluate the antibacterial activity of IgY, killing curves were performed. Results show that anti-ETEC IgY inhibited the growth of ETEC K88 in a dose-dependent manner (**Figure 1**). Moreover, the bacteriostatic effect was enhanced with the increase of anti-ETEC IgY concentration, which agrees with previous reports (Zhen et al., 2008; Sui et al., 2011).

### IgY Inhibited ETEC Adhesion and Damaged the Integrity of ETEC Cells

**Figure 2:**
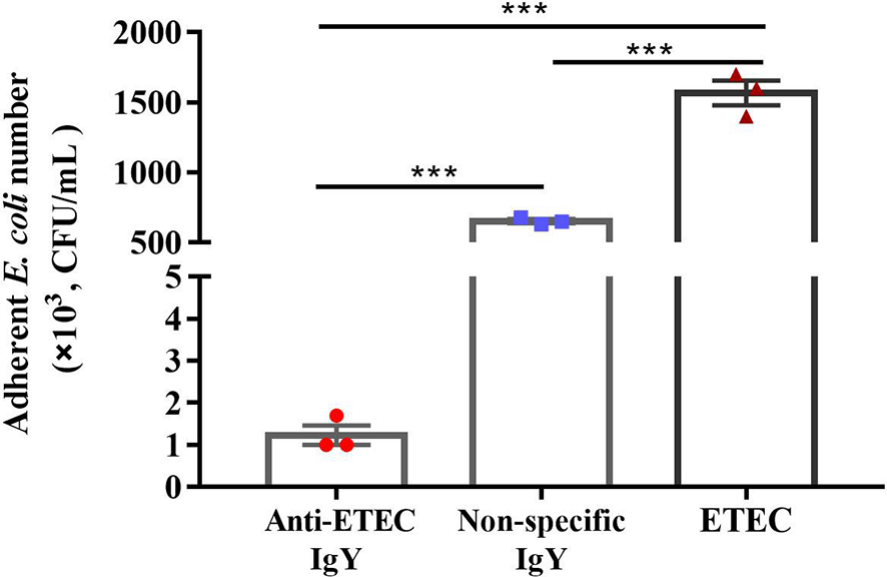
Inhibitory effect of IgY on adhesion of ETEC K88 to IPEC-J2 cells. Before infection, ETEC K88 (2 × 10^8^ CFU/mL) bacteria were pre-incubated with 5 mg/mL of anti-ETEC IgY, 5 mg/mL non-specific IgY or PBS at 37°C for 1 h, respectively. ETEC K88 bounded to IPEC-J2 cells were plated onto MacConkey-agar plated. Initial and final bacterial colonies were counted. Values are expressed as mean ± SEM (n = 3). ***P < 0.001.

**Figure 3:**
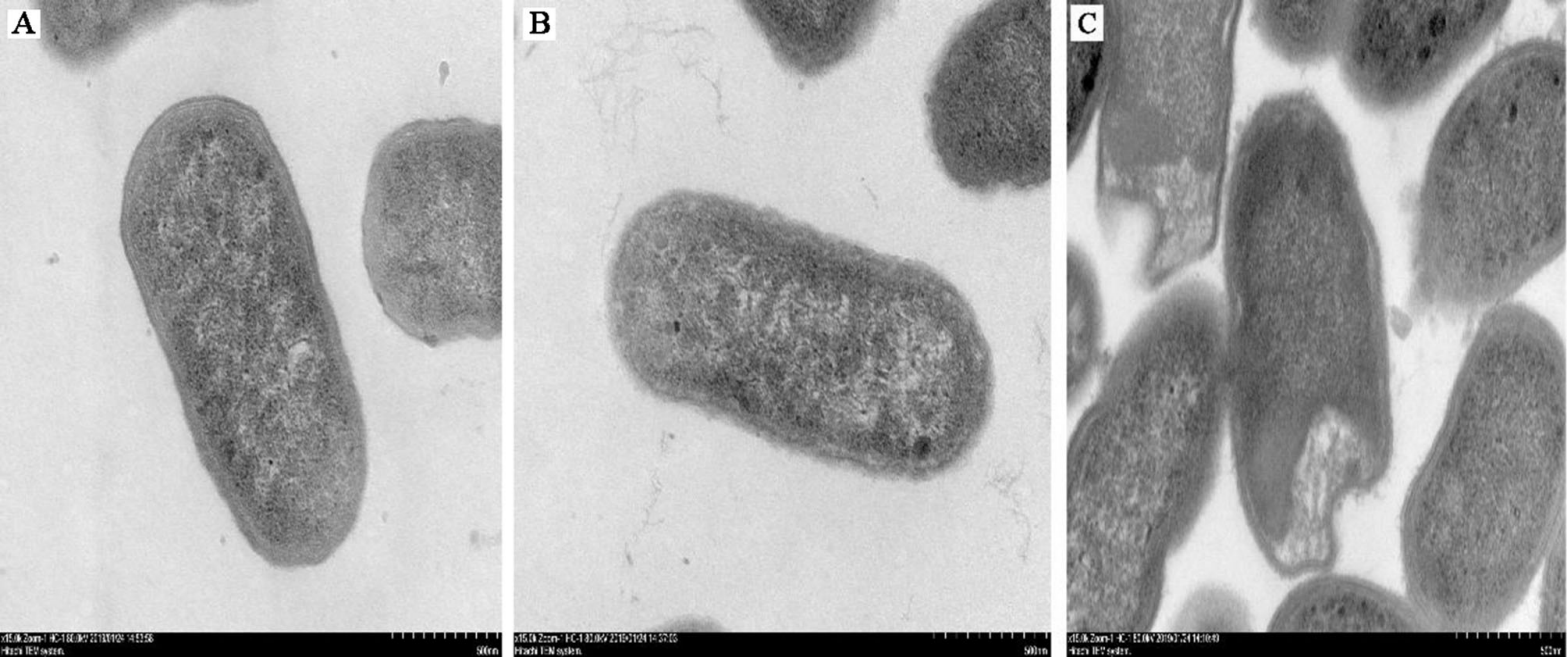
Micrographs of ETEC K88 cells (ultra-thin sections) after treatment. **(A)** ETEC K88 cells (10^7^ CFU/mL) were incubated with sterile PBS (0.1 mol/L, pH 7.4); **(B)** ETEC K88 cells (10^7^ CFU/mL) were incubated with 5 mg/mL of non-specific IgY; **(C)** ETEC K88 cells (10^7^ CFU/mL) were incubated with 5 mg/mL of anti-ETEC IgY.

To determine whether IgY could inhibit bacterial adhesion and damage the cell morphology and integrity of ETEC, adherence inhibition and TEM assay were performed. Results showed that, compared with the ETEC infection group, anti-ETEC IgY and non-specific IgY decreased adhesion of ETEC K88 to IPEC-J2 cells (*P* < 0.05), and anti-ETEC IgY expressed greater antibacterial activity than non-specific IgY (**Figure 2**). ETEC K88 cells in the control and non-specific IgY groups had an intact morphology and a smooth surface. However, after treatment with anti-ETEC IgY, some ETEC K88 cell walls were thinner, blurred, or even missing (**Figure 3**).

### IgY Ameliorated ETEC-Induced Clinical Signs

**Figure 4:**
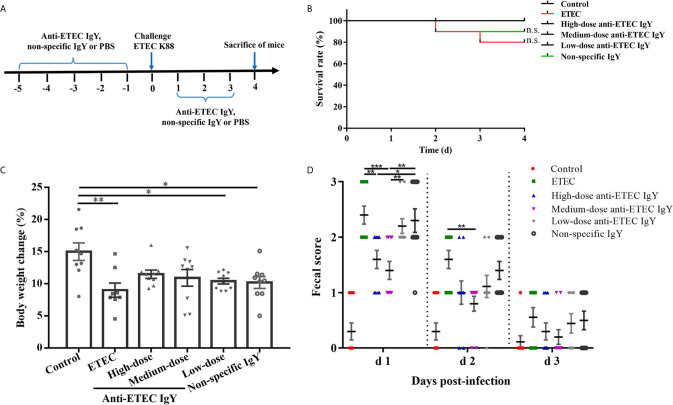
Effects of IgY on clinical signs of ETEC-infected mice. **(A)** Schematic diagram of the experimental design for the protective effects of anti-ETEC K88 IgY on ETEC infection in mice. **(B)** Survival rate of control (neither IgY nor ETEC K88 infection) mice, ETEC-infected mice, ETEC-infected mice treated with high-, medium- or low-doses of anti-ETEC K88 IgY, or ETEC-infected mice treated with non-specific IgY (n=10/group). **(C)** Body weight change of control mice, ETEC-infected mice, mice treated with high-dose, medium-dose or low-doses anti-ETEC IgY or non-specific IgY groups (n=9 except n=8 for ETEC group). **(D)** Fecal score on different days after ETEC K88 infection in mice (d1, n=10; d2, n=10; d3, n=9). Fecal scoring scale was performed from 0 to 3 (0, dry stool; 1, slight sticky stool; 2, soft stool; 3, liquid stool). Values reported are least squares mean ± SEM. *P < 0.05, **P < 0.01, ***P < 0.001, ns, not significant.

The schematic diagram of the experimental design for the protective effects of anti-ETEC IgY on mice infected with ETEC K88 is shown in **Figure 4A**. To make a risk assessment of IgY and investigate whether anti-ETEC IgY administrated orally could relieve negative effects induced by ETEC in mice, we firstly monitored clinical signs of mice daily. After ETEC K88 challenge, mice showed different degrees of clinical signs, which included lethargy, appetite loss, curled up body posture, shivering, and soft stools, while mice in the control group did not exhibit any clinical signs of ETEC infection. No significant difference in survival rates was observed among treatments (*P* > 0.05; **Figure 4B**). Mice in ETEC K88-infected group had lower body weight gains (*P* < 0.05; **Figure 4C**) and higher fecal scores at day 1 and day 2 post-infection (*P* < 0.05; **Figure 4D**) than control mice. However, mice pretreated with high- and medium-dose anti-ETEC IgY attenuated the reduction of body weight and were similar with control mice (*P* > 0.05). On day 1 post infection, fecal scores of mice in both high- and medium-dose anti-ETEC IgY groups were lower than in the ETEC K88 and non-specific IgY groups (*P* < 0.05). On day 2 post infection, the fecal score of mice in medium-dose anti-ETEC IgY group was lower than in the ETEC K88 group (*P* < 0.05). Results indicated that high-, and medium-dose anti-ETEC IgY could ameliorated ETEC-induced clinical signs more effectively.

### IgY Inhibited ETEC Colonization and Improved SCFA Concentrations

**Figure 5:**
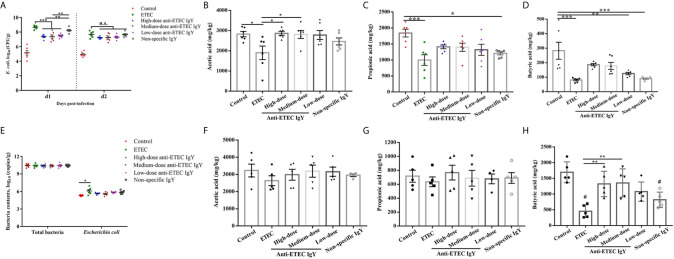
Effects of IgY on bacterial load and microbial metabolites in feces and cecal digesta of ETEC-infected mice. **(A)** *E. coli* number in feces of mice on different days post-infection. Mice in ETEC group were challenged with ETEC and treated with PBS. Mice in IgY groups were pre-treated with high-, medium- or low-doses anti-ETEC IgY or non-specific IgY and then challenged with ETEC, n=6 **(B–D)** Short chain fatty acid (acetic acid, propionic acid, and butyric acid) concentration in feces of mice, n=6. **(E)** Total bacteria and *E. coli* content in cecal digesta of mice at day 4 post-infection, n=6. **(F–H)** Short chain fatty acids (acetic acid, propionic acid and butyric acid) concentrations in cecal digesta of mice, n=5. Values are expressed as least squares mean ± SEM, *P < 0.05, **P < 0.01, ***P < 0.001. ns, not significant. ^#^P <0.05 compared to control group.

To study whether this positive effects of anti-ETEC IgY on clinical signs of challenged mice was caused by a difference in bacteria removal efficiency, the viable cell counts of *E. coli* in fresh feces of mice from day 1 and 2 after infection were measured. On day 1 post infection, compared with control group, the numbers of *E. coli* in feces of all challenged mice were increased (*P* < 0.05; **Figure 5A**). Numbers of *E. coli* in feces of mice treated with different doses of anti-ETEC IgY decreased compared with the ETEC-infected group (*P* < 0.05), and numbers of *E. coli* in feces of mice treated with high- and medium-dose anti-ETEC IgY were lower than the non-specific IgY group (*P* < 0.05). On day 2 post infection, no significant differences were observed in the number of *E. coli* in feces among anti-ETEC IgY treated and ETEC-infected groups.

In terms of SCFAs, compared with the control group, ETEC K88 infection decreased (*P* < 0.05) the concentrations of acetic acid, propionic acid, and butyric acid in the feces of mice infected with ETEC (*P* < 0.05; **Figures 5B–D**). Similarly, propionic acid and butyric acid contents in the feces of infected mice treated with non-specific IgY were decreased compared with controls. Acetic acid contents in feces of infected mice treated with high- and medium-dose anti-ETEC IgY were increased compared with the ETEC-infected group. Moreover, the concentrations of SCFA in feces of mice treated with high- and medium-dose anti-ETEC IgY returned to the normal levels in the control group.

The amount of *E. coli* in cecal contents of ETEC-infected mice increased (*P* < 0.05) compared with control. However, oral gavage with IgY partially mitigated the bacteria load and were similar to control (**Figure 5E**). Concentrations of butyric acid were decreased in cecal contents of ETEC-infected mice and infected mice treated with non-specific IgY compared with the control group (*P* < 0.05; **Figure 5H**). Mice treated with high- and medium-dose anti-ETEC IgY had greater butyric acid levels in cecal contents compared with ETEC-infected mice (*P* < 0.05). No differences were observed on the concentrations of acetic acid and propionic acid among treatments (**Figures 5F, G**). These results indicated that anti-ETEC IgY improved bacteria removal efficiency, and positively affected the microbial metabolites.

### Effects of IgY on Serum Biochemical Indices and Immunoglobulins

To evaluate the effects of different doses of anti-ETEC IgY on liver function and immunity in ETEC-infected mice, we determined serum indicators. As shown in **Table 1**, compared with the control group, serum globulin and aspartate transaminase (AST) contents increased, and albumin to globulin ratio decreased in ETEC-infected mice, and serum AST contents increased in mice treated with non-specific IgY group (*P* < 0.05). Serum AST level in mice treated with medium-dose anti-ETEC IgY was lower than that in ETEC-infected mice (*P <*0.05), and similar to the normal levels of the control group.

**Table 1:** Effects of IgY on serum biochemical parameters of mice challenged by ETEC K88.

Item	Control	ETEC challenge	Levels of anti-ETEC IgY(mg/mL)			Non-specific IgY	SEM	*P* value
			32	16	8			
Total protein (g/L)	58.70	61.88	60.40	57.50	57.50	59.63	1.81	0.51
Albumin (g/L)	33.90^a^	30.38^ab^	32.40^ab^	29.50^b^	31.30^ab^	29.50^b^	0.95	0.02
Globulin (g/L)	24.80^b^	31.50^a^	28.00^ab^	28.00^ab^	26.20^ab^	30.13^ab^	1.27	0.02
Albumin to globulin ratio	1.38^a^	0.96^b^	1.17^ab^	1.12^ab^	1.21^ab^	0.98^b^	0.06	< 0.01
Alanine aminotransferase (U/L)	53.63	82.80	75.30	76.40	66.75	74.90	7.70	0.19
Aspartate transferase (U/L)	124.10^c^	194.13^a^	164.50^abc^	138.63^bc^	170.20^ab^	171.90^ab^	10.77	< 0.01
Urea nitrogen (mmol/L)	8.85	10.49	9.04	8.99	9.01	9.08	0.67	0.56

^a-c^Values with different superscripts within a row differ (P < 0.05), n=6. Mice in control group were not challenged with ETEC K88. Mice in ETEC group were challenged with ETEC K88 and treated with PBS. Mice in IgY groups were challenged with ETEC K88 and treated with 250 μL of high-, medium- or low-doses anti-ETEC IgY or 250 μL of non-specific IgY.

Compared with the control group, ETEC K88 infection increased serum immunoglobulin IgG and IgA concentrations in mice (*P* < 0.05; **Table 2**). Serum IgG concentrations in infected mice treated with anti-ETEC IgY were decreased (*P* < 0.05) compared with ETEC-infected mice and were similar to the normal levels of the control mice. Serum IgA concentrations in infected mice treated with high- and medium-dose anti-ETEC IgY were lower than in ETEC-infected mice and were not different from control mice. Concentration of sIgA in jejunum of mice treated with medium-dose anti-ETEC IgY was increased compared with ETEC-infected mice and infected mice treated with non-specific IgY (*P* < 0.05). Results suggested that ETEC could reduce liver damage and immune overreaction, but this phenomenon was partly recovered by high- or medium-dose anti-ETEC IgY.

**Table 2:** Effects of IgY on serum immunoglobulins and sIgA level in jejunum of mice challenged by ETEC K88.

Immunoglobulin	Control	ETEC challenge	Levels of anti-ETEC IgY(mg/mL)			Non-specific IgY	SEM	*P* value
			32	16	8			
IgG, μg/mL	312.29^b^	401.28^a^	333.56^b^	350.20^b^	340.34^b^	394.71^a^	9.46	< 0.01
IgM, μg/mL	48.50	52.27	48.36	50.43	47.10	47.00	3.27	0.86
IgA, ng/mL	460.33^c^	543.04^a^	485.42^bc^	463.43^c^	510.40^ab^	539.71^a^	9.47	< 0.01
sIgA, μg/mL	12.22^c^	13.22^bc^	14.54^ab^	15.35^a^	13.90^b^	13.64^bc^	0.31	< 0.01

^a-c^Values with different superscripts within a row differ (P < 0.05), n=6. Mice in control group were not challenged with ETEC K88. Mice in ETEC group were challenged with ETEC K88 and treated with PBS. Mice in IgY groups were challenged with ETEC K88 and treated with 250 μL of high-, medium- or low-doses of anti-ETEC IgY or 250 μL of non-specific IgY.

### IgY Ameliorated ETEC-Induced Intestinal Inflammation and Permeability

To test the anti-inflammatory effects of IgY on ETEC-induced enteritis, we evaluated the secretion and mRNA expression of inflammatory cytokines. Compared with the control group, the concentration of IL-1β in serum of mice in ETEC-infected group increased (*P* < 0.05), and concentration of IL-4 decreased (*P* < 0.05; **Table 3**). Mice treated with different doses of anti-ETEC IgY had lower IFN-γ and IL-1β concentrations in serum than in ETEC-infected mice (*P* < 0.05). Concentrations of IL-1β were lower than that of mice treated with non-specific IgY (*P* < 0.05). Serum IL-10 content of mice treated with medium-dose anti-ETEC IgY was higher and KC/GRO content was lower than mice in the ETEC-infected group (*P* < 0.05), and that were similar to the control mice.

**Table 3:** Effects of IgY on serum cytokines of mice challenged by ETEC K88.

Cytokines	Control	ETEC challenge	Levels of anti-ETEC IgY(mg/mL)			Non-specific IgY	SEM	*P* value
			32	16	8			
IFN-γ	1.51^ab^	2.00^a^	1.12^b^	1.05^b^	0.95^b^	1.32^ab^	0.17	< 0.01
IL-10	16.96^ab^	12.95^b^	19.15^ab^	20.42^a^	18.06^ab^	17.78^ab^	1.53	0.05
IL-12p70	20.03^ab^	22.28^a^	14.80^b^	16.73^ab^	16.00^ab^	15.89^ab^	1.48	0.01
IL-1β	7.62^bc^	13.16^a^	6.19^c^	4.89^c^	4.50^c^	10.81^ab^	0.87	< 0.01
IL-2	3.15	2.98	3.01	2.94	2.19	2.43	0.40	0.47
IL-4	0.63^a^	0.40^b^	0.47^ab^	0.48^ab^	0.41^b^	0.43^b^	0.04	< 0.01
IL-5	6.27	4.27	4.93	4.32	4.46	4.43	0.64	0.27
IL-6	10.40	7.08	8.93	10.57	12.18	8.97	2.04	0.58
KC/GRO	40.35^ab^	57.01^a^	43.57^ab^	38.03^b^	38.97^ab^	52.38^ab^	3.94	0.02
TNF-α	15.72^ab^	20.56^a^	16.59^ab^	11.88^b^	12.68^b^	16.50^ab^	1.33	< 0.01

^a-c^Values with different superscripts within a row differ (P < 0.05), n=5. Mice in control group were not challenged with ETEC K88. Mice in ETEC group were challenged with ETEC K88 and treated with PBS. Mice in IgY groups were infected with ETEC K88 and treated with 250 μL of high-, medium- or low-doses anti-ETEC IgY or 250 μL of non-specific IgY.

**Figure 6:**
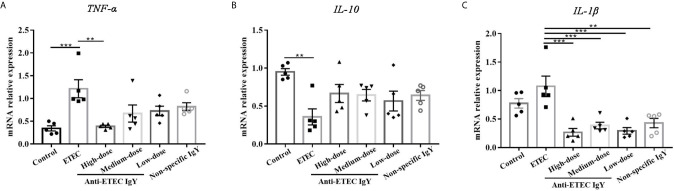
Proinflammatory and anti-inflammatory cytokines mRNA expression in jejunum of control (neither IgY nor ETEC infection) mice, ETEC-infected mice, ETEC-infected mice treated with high-, medium- or low-doses anti-ETEC IgY or ETEC-infected mice treated with non-specific IgY. **(A)** TNF-α mRNA expression; **(B)** IL-10 mRNA expression; **(C)** IL-1β mRNA expression. Values are expressed as least squares mean ± SEM, n =5. **P < 0.01, ***P < 0.001.

Consistent with the results of cytokines in serum, ETEC-infected mice had a greater mRNA relative expression of *TNF-α*, and a lower mRNA relative expression of *IL-10* in jejunum than those in control mice (**Figures 6A, B**). Fortunately, compared with ETEC-infected mice, mice treated with high-dose anti-ETEC IgY decreased *TNF-α* mRNA relative expression (*P* < 0.05; **Figure 6A**). Specific and non-specific IgY all reduced *IL-1β* mRNA relative expression in jejunum of mice (*P* < 0.05; **Figure 6C**). These results indicated that oral administration of ETEC specific IgY is efficacious in reducing intestinal inflammation associated with ETEC infection in mice.

**Figure 7:**
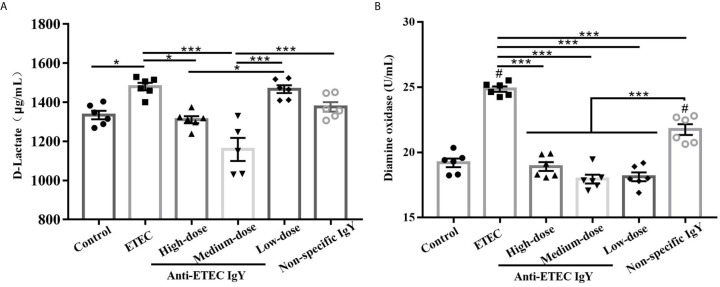
D-Lactate **(A)** and diamine oxidase **(B)** contents in serum of control (neither IgY nor ETEC infection) mice, ETEC-infected mice, ETEC-infected mice treated with high-, medium- or low-doses anti-ETEC IgY or ETEC-infected mice treated with non-specific IgY. Values are expressed as least squares mean ± SEM, n =6. *P < 0.05, ***P < 0.001. ^#^ *P <*0.05 compared to control group.

Following inflammation, we investigated whether IgY could ameliorate ETEC-induced intestinal permeability. Results showed that concentrations of D-lactate (**Figure 7A**) and DAO (**Figure 7B**) in serum of mice in the ETEC-infected group were higher than mice in the control group (*P* < 0.05), while the increases were limited in those from high- and medium-dose anti-ETEC IgY groups (*P* < 0.05). Compared with the non-specific IgY group, anti-ETEC IgY could reduce intestinal permeability more effectively. Taken together, these results demonstrated that prophylactic administration of anti-ETEC IgY relieves intestinal inflammation and permeability induced by ETEC.

### IgY Improved ETEC-Induced Intestinal Morphological Injury

To further evaluate the status of intestinal injury risk, intestinal morphological analysis and histopathological scores were performed. Villus height and V/C ratio decreased and the crypt depth increased in the jejunum and ileum of the ETEC-infected mice compared with controls (*P* < 0.05; **Table 4**). Oral administration of different doses of IgY improved jejunum and ileum tissue morphology of challenged mice in varying degrees. Mice treated with high and medium-doses anti-ETEC IgY had higher villi height and V/C ratio in jejunum and ileum than mice in ETEC-infected group (*P* < 0.05), and were not different from control mice.

**Table 4:** Effect of IgY on the morphology of jejunum and ileum in ETEC K88 infected mice.

Item	Control	ETEC challenge	Levels of anti-ETEC IgY (mg/mL)			Non-specific IgY	SEM	*P*-value
			32	16	8			
Jejunum								
Villus height, μm Crypt depth, μm Villus height/crypt depth	404^a^	271^b^	404^a^	376^a^	342^a^	349^a^	16	< 0.01
	93^c^	123^a^	103^bc^	110^abc^	108^abc^	114^ab^	5	< 0.01
	4.37^a^	2.22^c^	3.98^ab^	3.49^ab^	3.21^bc^	3.08^bc^	0.22	< 0.01
Ileum								
Villus height, μm Crypt depth, μm Villus height/crypt depth	295^a^	157^b^	283^a^	264^a^	253^a^	223^ab^	20	< 0.01
	99^b^	146^a^	108^b^	108^b^	114^b^	115^b^	6	< 0.01
	3.00^a^	1.10^c^	2.64^ab^	2.49^ab^	2.22^ab^	1.97^bc^	0.22	< 0.01

^a-c^Values with different superscripts within a row differ (P < 0.05), n=6. Mice in control group were not challenged with ETEC K88. Mice in ETEC group were challenged with ETEC K88 and treated with PBS. Mice in IgY groups were challenged with ETEC K88 and treated with 250 μL of high-, medium- or low-doses anti-ETEC IgY or 250 μL of non-specific IgY.

**Figure 8:**
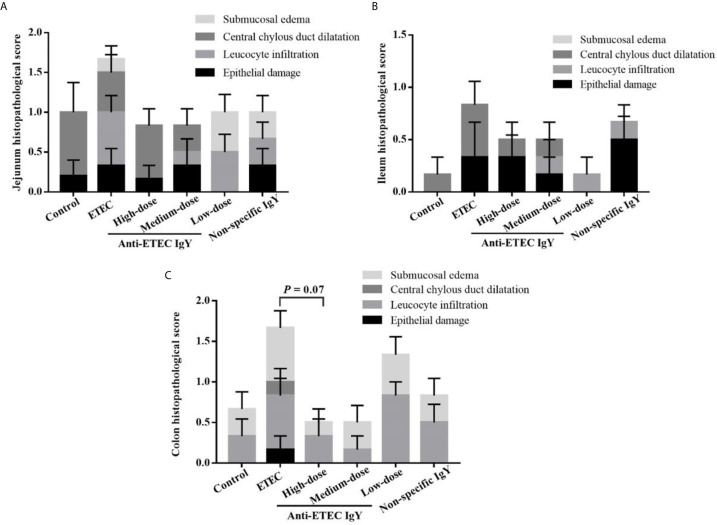
Pathological score of jejunum **(A)**, ileum **(B)** and colon **(C)** tissues in control (neither IgY nor ETEC K88 infection) mice, ETEC-infected mice, ETEC-infected mice treated with 250 μL of high-dose (32 mg/mL), medium-dose (16 mg/mL) or low-dose (8 mg/mL) anti-ETEC K88 IgY, or ETEC-infected mice treated with 250 μL of non-specific IgY (16 mg/mL). Figures are four different graphs laid on top of each other. Pathological scores were assessed according to the following degrees: 0, none;1, mild; 2, moderate; 3, severe. Data are presented as least squares mean ± SEM, n=6.

Infection with ETEC K88 caused the jejunum, ileum and colon of mice to present submucosal edema, central chylous duct dilatation, leucocyte infiltration and epithelial damage (**Figures 8A–C**). Oral administration of the high dose of anti-ETEC IgY tended to reduce total colon histopathological score of mice (*P* = 0.07) compared with ETEC-infected group.

### IgY Positively Modulated Intestinal Mucosal Immune Response

**Figure 9:**
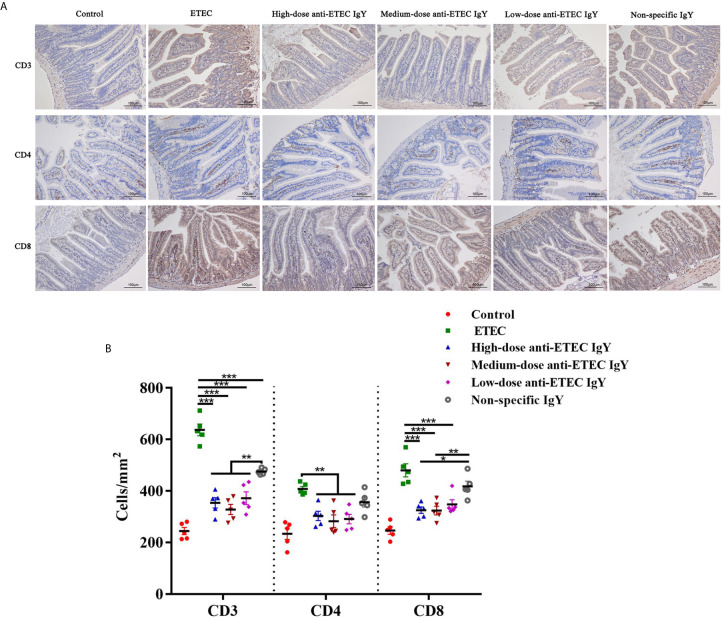
**(A)** Representative images of the immunohistochemical localization of lymphocytes in the jejunum of control (neither IgY nor ETEC infection) mice, ETEC-infected mice, ETEC-infected mice treated with high-, medium- or low-doses anti-ETEC IgY or ETEC-infected mice treated with non-specific IgY. The lymphocyte markers and nuclei are stained brown and blue, respectively. **(B)** Quantification of lymphocyte populations in the jejunum of mice in different groups. Values are least squares means ± SEM, n=5. *P < 0.05, **P < 0.01, ***P < 0.001.

To further determine how IgY modulates immune response of ETEC-infected mice, we analyzed the effects of IgY on lymphocytes in jejunum. Different types of jejunal lymphocytes were labeled, localized, and counted and different lymphocyte populations were shown in brown area (**Figure 9A**). ETEC K88 challenge increased number of T lymphocytes (CD3^+^), T helper lymphocytes (CD4^+^) and T suppressor/cytotoxic lymphocytes (CD8^+^) compared with control group (*P* < 0.05; **Figure 9B**). Different doses of anti-ETEC IgY all attenuated the increase in the number of T lymphocytes (CD3^+^, CD4^+^ and CD8^+^). Non-specific IgY attenuated the increase in number of CD3^+^ lymphocytes induced by ETEC K88 challenge (*P* < 0.05). Compared with the non-specific IgY group, high and medium-doses anti-ETEC IgY inhibited the increase in number of CD3^+^ and CD8^+^ lymphocytes (*P* < 0.05), and CD8^+^ lymphocytes concentrations nearly returned to the normal level of the control group.

## Discussion

ETEC is one of the pathogens that causes moderate-to-severe intestinal inflammation, diarrhea, and even death among young animals and human infants (Mondal et al., 2009; Shah et al., 2009). In the current study, we investigated the bacteriostatic activity of IgY against ETEC K88 *in vitro*, and evaluated the protective effects of anti-ETEC against ETEC infection *in vivo*. Results suggested that oral administration of anti-ETEC specific IgY is efficacious in reducing morbidity and intestinal inflammation associated with ETEC infection in mice.

Experiments *in vitro* showed that anti-ETEC IgY could cause agglutination of bacteria and effectively inhibit the growth of ETEC K88, reduce the adherence of ETEC K88 to IPEC-J2 cells, and destroy the integrity of the ETEC K88 bacterial cell wall. These findings are consistent with Lee et al. (2002), who found that anti-*Salmonella* IgY can bind to antigens expressed on the surface of *Salmonella*, resulting in surface structure alterations of the bacteria. These findings may contribute to why specific IgY antibody acts as a bacteriostatic factor in the absence of other factors such as serum complement.

To evaluate the preventive effect of IgY against ETEC K88 infection, we established a mouse model of intestinal inflammation induced by ETEC K88 similar to that reported previously (Yu et al., 2020). Mice exhibited typical clinical signs of ETEC infection which included curled up body posture, shivering, diarrhea, weight loss, intestinal inflammation, and death, which indicates that this model can be used to evaluate the efficacy of IgY against ETEC K88 infection. Both high and medium-doses anti-ETEC IgY effectively relieved the weight loss and the severity of diarrhea. This is in accord with a previous report, that found anti-*S. typhimurium* IgY provided effective protection against *S. typhimurium*-induced intestinal infection (Li et al., 2016). Results in this study suggest that oral administration of specific IgY was able to improve clinical signs.

Gut microbiota plays an important role in maintaining intestinal microecological balance. Fecal *E. coli* number of mice infected with ETEC K88 increased sharply, but mice treated with anti-ETEC IgY reduced fecal *E. coli* load on day 1 post infection. Similarly, Rahimi et al. (2007) found that IgY against *Salmonella enteritis* could reduce excretion of *Salmonella* in the feces of *Salmonella*-infected laying hens, and reduce its colonization in hen’s cecum. Other researchers (Yi et al., 2017) reported that infecting mice with *E. coli* O157:H7 increased the number of *E. coli* and decreased the number of *Lactobacillus* in cecum. Results suggest that anti-ETEC IgY can promote pathogen excretion, thereby reducing their colonization in mice intestine. Increased bacteria removal efficiency may contribute to the positive effects of anti-ETEC IgY on clinical signs of challenged mice.

Metabolites of intestinal bacteria are vital to maintain integrity of the intestinal barrier function. SCFAs produced by intestinal microbial fermentation of carbohydrates have diverse functional roles in various physiologic processes (Koh et al., 2016). In the present study, ETEC K88 infection reduced levels of acetic acid, propionic acid, and butyric acid in feces of mice. High- and medium-doses anti-ETEC IgY were more effective than low dose of anti-ETEC IgY and non-specific IgY at restoring SCFA concentrations. These results imply that anti-ETEC IgY can effectively improve metabolism of intestinal microbiota.

We also investigated effects of IgY on biochemical indices in serum of ETEC K88 challenged mice. ALB plays a vital role in maintaining normal osmotic pressure of blood and ensures nutrient supply to the body. Liver is rich in enzymes that are involved in protein synthesis and catabolism. ALT and AST in serum are important transaminases and often used as an enzymatic index to evaluate liver function (Zhu et al., 2016). When the liver has pathological changes or injuries, concentration of GLB, ALT and AST in serum increased. We found that ETEC K88 challenge significantly increased GLB content and decreased the ratio of ALB to GLB in serum. Oral gavage with anti-ETEC IgY partially mitigated the increase of GLB and AST and the decrease of the ratio of ALB to GLB induced by ETEC K88 challenge. These findings suggest that anti-ETEC IgY can partially relieve liver damage of mice induced by ETEC K88.

Immunoglobulins are immune effector molecules that play an important role in resisting the infection of external pathogens and can reflect immune level of the body. A previous study demonstrated that serum IgG, IgA and IgM levels significantly increased in piglets challenged with *Salmonella* or *E. coli* (Szabó et al., 2008). In the present study, we found that IgA and IgG content in serum of ETEC-infected mice increased significantly compared with non-infected mice. Oral gavage with high- and medium-doses anti-ETEC IgY effectively mitigated the increased IgA and IgG secretion caused by ETEC K88 challenge. In addition, sIgA plays an important role in antibody-mediated humoral immunity and maintaining intestinal mucosal homeostasis by binding to pathogenic bacteria which prevents bacterial invasion and adhesion (Fagarasan, 2008). The sIgA content of the jejunum in mice treated with anti-ETEC IgY increased in varying degrees. Similarly, Liu et al. (2017) found that ETEC infection decreased mRNA expression of the polymeric immunoglobulin receptor involved in sIgA transport in mouse intestinal epithelial cells, which increased contents of sIgA in jejunum and ileum of mice after challenge. This observation was consistent with the trend of sIgA content in jejunum of ETEC-infected mice in our study. These data suggested that anti-ETEC IgY modulated the body’s inflammatory response and promoted pathogen excretion in ETEC K88 infected mice.

ETEC infection activates the body’s immune system. The immune response elicits an increase in IL-1β, TNF-α, IL-6 and other pro-inflammatory cytokines to resist invasion of exogenous pathogens which produces on inflammatory response. Excessive expression of pro-inflammatory cytokines seriously damages health of the body (Yu et al., 2018). In this study, ETEC K88 challenge increased the secretion of pro-inflammatory cytokine IL-1β and the expression of *TNF-α* and *IL-1β*, decreased the secretion of anti-inflammatory cytokine IL-4 and the expression of *IL-10* in jejunum of mice. This may be due to higher levels of pro-inflammatory cytokines that inhibited anti-inflammatory cytokine production in mice, in a negative feedback regulation of the immune system (Gao et al., 2013). Our study found that mice pretreated with specific IgY effectively inhibited ETEC-induced inflammatory response.

Maintaining integrity of the intestinal epithelial barrier is very important for defense against pathogen invasion and inflammation (Fasano and Shea-Donohue, 2005; Liu et al., 2017). Serum D-lactate and DAO have been used as markers for reflecting intestinal permeability and barrier function (Wijtten et al., 2011). In the present study, Anti-ETEC IgY effectively inhibited the increase of D-lactate and DAO levels in serum of mice compared with ETEC-infected mice. The high- and medium-doses anti-ETEC IgY were more effective than low-dose anti-ETEC IgY and non-specific IgY. Previous studies have reported that pathogens such as ETEC or *Salmonella* can increase concentration of D-lactate and DAO in serum and exacerbate the intestinal permeability (Yang et al., 2014; Xia et al., 2015). Our results indicate that anti-ETEC IgY improves the reduced intestinal permeability and enhances intestinal barrier function in ETEC K88 infected mice, which is consistent with our previous study (Han et al., 2019).

Intestinal morphology is an important indicator to evaluate digestive and absorptive functions of the gut. ETEC causes intestinal inflammation, and damages to intestinal tissues that results in bacterial invasion and enteric infection (Johnson et al., 2010). In the present study, ETEC K88 infection caused damage to the jejunum and ileum. Oral administration of different doses of anti-ETEC IgY reversed these negative impacts to varying degrees. This may be due to the preventive effects of anti-ETEC IgY in decreasing intestinal permeability and inflammatory responses. These results suggest that anti-ETEC IgY effectively relieve villous atrophy and crypt hyperplasia of jejunal and ileal tissue, thus improving intestinal tissue morphology in mice challenged with ETEC K88.

To further investigate potential mechanisms through which IgY exert protection, we also surveyed effects of IgY on the mucosal immune response during ETEC K88 infection. T lymphocytes in intestines are composed of two main subpopulations, namely CD4^+^ lymphocytes (T helper lymphocytes) and CD8^+^ lymphocytes (T suppressor/cytotoxic lymphocytes). Pathogen infection can mobilize intestinal mucosal immune responses to defend against the pathogen invasion. Previous reports have shown that bioactive substances including fructo-oligosaccharide and spray-dried porcine plasma (containing immunoglobulin G) can regulate the immune response of intestinal-associated lymphoid tissue in animals (Pérez-Bosque et al., 2004; Watzl et al., 2005). Another study showed that IgY modulates the immune response against bovine rotavirus infection at the mucosal level in newborn calves (Vega et al., 2011), but the immuno-modulatory functions of IgY during infection are still unclear. In our study, ETEC K88 challenge increased T lymphocytes (CD3^+^), T helper lymphocytes (CD4^+^) and T suppressor/cytotoxic lymphocytes (CD8^+^) in the jejunum of mice, that probably due to ETEC K88 excessive stimulated the mucosal immune system. Nevertheless, anti-ETEC IgY notably inhibited excessive proliferation of jejunal T lymphocytes caused by ETEC K88 infection. Compared with non-specific IgY, anti-ETEC IgY was more effective in regulating the immune response of intestinal mucosa. This effect is similar with that observed by Li et al. (2016) who found that IgY played an important immuno-modulatory role in mice challenged with *S. typhimurium*. Hence, the data suggest that IgY can alleviate excessive immune stresses induced by ETEC K88 infection, so that homeostasis of the intestinal environment is maintained.

In summary, we successfully established an ETEC-infected mouse model of intestinal inflammation. We found that specific IgY exhibited strong anti-infective activity *in vitro*, and effectively ameliorated the ETEC K88-induced inflammation response, mucosal morphology damage and intestinal epithelial barrier injury. Anti-ETEC IgY decreased fecal and cecal bacterial load, and modulated the intestinal mucosal immune response. In addition, the overall effects of high- (32 mg/mL) and medium- (16 mg/mL) dose anti-ETEC IgY were more effective than low- (8 mg/mL) dose anti-ETEC IgY and non-specific IgY. Our results suggest that anti-ETEC IgY has a positive effect in improving intestinal health and regulating immune response. Consequently, anti-ETEC IgY may be a prophylactic immunotherapy agent against infection of intestinal tissues by pathogenic bacteria.

## Data Availability Statement

The raw data supporting the conclusions of this article will be made available by the authors, without undue reservation.

## Ethics Statement

The animal study was reviewed and approved by Institutional Animal Care and Use Committee at China Agricultural University.

## Author Contributions

SH: Conceptualization, methodology, validation, formal analysis, investigation, data curation and writing-original draft preparation. YW: Conceptualization, methodology and formal analysis. FY: Validation and formal analysis. PH: Conceptualization, writing-review and editing, supervision, project administration and funding acquisition. All authors contributed to the article and approved the submitted version.

## Funding

The authors declare that all sources of funding received for the research have been submitted.

## Conflict of Interest

Author FY was employed by Hubei Shendi Biological Technology Co., LTD.

The remaining authors declare that the research was conducted in the absence of any commercial or financial relationships that could be construed as a potential conflict of interest.
