# Supplementary material for: Chicken Egg Yolk Antibody (IgY) Protects Mice Against Enterotoxigenic Escherichia coli Infection Through Improving Intestinal Health and Immune Response
Source: Front Cell Infect Microbiol. 2021 Apr 13;11:662710. doi: 10.3389/fcimb.2021.662710 (PMC8076637; doi:10.3389/fcimb.2021.662710)
Supplement: Supplementary file 1 [file Table_1.docx]

**TABLE S1 | The primer sequences for RT-qPCR**

| Genes | Orientation | Sequence 5'-3' | Size (bp) |
| --- | --- | --- | --- |
| *β-actin* | Forward | TCCATCATGAAGTGTGACGT | 154 |
|  | Reverse | GAGCAATGATCTTGATCTTCAT |  |
| *TNF-α* | Forward | TAACTTAGAAAGGGGATTATGGCT | 264 |
|  | Reverse | TGGAAAGGTCTGAAGGTAGGAA |  |
| *IL-10* | Forward | ACAACATACTGCTAACCGACTCCT | 239 |
|  | Reverse | CTGCTCCACTGCCTTGCTCTT |  |
| *IL-1β* | Forward | AAAGCCTCGTGCTGTCGG | 511 |
|  | Reverse | TAAGGAGTCCCCTGGAGATTG |  |
